# Promoting Physical Activity in Patients with Colon Adenomas: A Randomized Pilot Intervention Trial

**DOI:** 10.1371/journal.pone.0039719

**Published:** 2012-07-13

**Authors:** Kathleen Y. Wolin, Casey Fagin, Aimee S. James, Dayna S. Early

**Affiliations:** Washington University School of Medicine and Siteman Cancer Center, St Louis, Missouri, United States of America; University of Granada, Spain

## Abstract

**Background:**

Physical activity decreases risk of colon polyps and colon cancer and might reduce risk of colon cancer recurrence. Focusing on recent calls for translation of epidemiologic evidence into clinical care, our pilot study delivered an evidence-based physical activity intervention in adults with polyps, who are thus at elevated risk of developing colon cancer. The objective was to evaluate change in physical activity, measured by steps per day and minutes of moderate/vigorous physical activity.

**Methods:**

Sixteen adults with adenomas detected and removed at screening colonoscopy were recruited to a 12-week physical activity intervention. Participants were randomized to receive a standard (30 minutes/day) or high (60 minutes/day) walking program. Physical activity was measured via blinded pedometer and accelerometer at baseline and follow-up. Intervention messages focused on self-monitoring using pedometers and overcoming barriers to engaging in physical activity.

**Results:**

Participants in both arms significantly increased objectively measured minutes of moderate/vigorous physical activity over the course of the intervention. Both arms exceeded the intervention goal, but there was not a significant difference between arms at follow-up. Results were similar for pedometer measured physical activity, with a significant overall increase in steps/day from baseline to follow-up, but no between arm difference in change.

**Conclusion:**

Simple interventions of minimal contact time focusing on walking can significantly increase physical activity in individuals at increased risk of developing colon cancer.

**Trial Registration:**

ClinicalTrials.gov NCT01476631

## Introduction

Evidence linking regular physical activity with a reduced risk of colon cancer is consistent and convincing [1]–[3]. A recent meta-analysis of observational data found physical activity decreased risk of adenomatous polyps which are precursors to colon cancer [4]. There is no evidence of an association for physical activity with rectal cancer [2], [5]. Despite the benefits of physical activity, nearly 75% of the population fails to meet recommended physical activity levels [6]–[8].

Previous studies have reported a reduced risk of colon cancer with engagement in moderate intensity activity [9], [10]. Recent analyses in the Nurses’ Health Study, the largest prospective study to examine this association, found a significant risk reduction in colon cancer incidence among women walking at least two hours per week [11]. Observational data in colon cancer survivors shows a disease-free survival benefit for physical activity, but suggests that higher amounts of physical activity may be necessary to reduce risk of recurrence [12], [13]. Together, these data suggest there is a favorable role for physical activity in terms of risk reduction, at multiple stages in colon cancer carcinogenesis. However, there is little data on the role of physical activity in individuals at elevated risk of colon cancer, particularly those who have previously had colon adenomatous polyps. We therefore interpreted a need for data on whether a physical activity intervention could be successfully implemented after removal of adenomatous polyps during screening colonoscopy.

Designing such an intervention is challenging; the dose physical activity necessary to reduce risk of recurrent colon adenomas is unknown, as is whether increasing doses of physical activity would further modify the risk of adenoma recurrence. Data suggest that physical activity equivalent to 30 minutes of walking/day is adequate to reduce risk of developing colon cancer, while a higher exercise dose (60 min/day) may be necessary to reduce colon cancer recurrence and mortality. As physical activity interventions often struggle to achieve the intervention target dose, determining whether a higher dose is feasible is an important first step before broader dissemination or implementation of physical activity programs to prevent colon adenomas and cancer.

The overall objective of this study was to evaluate the feasibility of a pilot intervention to deliver two doses of physical activity delivered through an existing evidence-based walking intervention paradigm to individuals who have had colon polyps and are thus at increased risk for colon cancer. The First Step Program (FSP, also published as Manpo-Kei) is an evidence and theoretically-based two phase intervention that aims to promote uptake of and adherence to physical activity, specifically walking, using pedometers [14]–[22]. FSP addresses self-efficacy, outcome expectations and social support in line with social cognitive theory and moves participants through the phases of the Transtheoretical Model [23]. The intervention focuses on home-based moderate intensity walking with regular contact with study staff. FSP has repeatedly been shown to successfully increase physical activity in patient populations [16], [24], and in community settings [15], [22] Furthermore, the intervention successfully promoted a sustained increase in steps/day when implemented in “real world” settings, using existing diabetes educators and peer leaders [14] and in a community setting [21], indicating the intervention is effective and efficacious.

Pedometers are easy to use, relatively low cost, reliable and accurate [25]. The combination of the high frequency of walking as a physical activity and the comparatively low cost of pedometers has made them a popular tool for population-based research, both as a motivational and measurement device. Pedometer-based interventions typically focus on a 10,000 steps per day goal, which has support in clinical and monitored populations, [1], [2], [3] A review of 32 observational and intervention studies suggests that typical daily step counts range from: (1) 7–13,000 steps per day for healthy younger adults; (2) 6–8,500 steps per day for healthy older adults; and (3) 3,500–5,000 steps per day for sedentary individuals and those with disabilities of chronic illness [26]. These findings were corroborated in a study of urban African American adults [27], but data on other racial/ethnic groups has not yet been reported. Current research suggests step counts in the range of 3000–4000 steps are accumulated during 30-minutes of walking. [4], [5] Thus, for a healthy older adult, 10,000 steps would be accumulated through usual daily activities plus a 30-minute walk, making the 10,000 steps/day recommendation parallel to the physical activity guidelines [6] Reviews of walking interventions conclude that the use of pedometers [28] and telephone prompts [29], as is done in the second phase of FSP, successfully increase walking [30].

This study tested the feasibility of a pilot physical activity intervention designed for individuals with a recently resected colon adenoma. The focus of the study was on the development of an intervention that would require minimal face-to-face contact time to potentially improve future sustainability in clinical practice yet still initiate physical activity behavior change.

## Methods

In this study, we refined and pilot tested an evidence-based intervention to promote walking among individuals with a previous colon adenoma.

The Step Down Colon Cancer (SDCC) pilot tested an evidence-based intervention to promote walking, using pedometers, among individuals with a previous colon polyp (Protocol S1, Checklist S1). SDCC is a 12-week two-arm randomized controlled trial program prescribing two different doses (30 minutes vs 60 minutes) of walking-based physical activity [17], [31]. This study builds on previous physical activity interventions with cancer outcomes where 12 week interventions are common [32]–[36].

### Recruitment

To identify eligible patients, staff in the hospital-based gastroenterology practice reviewed practice records to identify individuals potentially meeting preliminary eligibility criteria. Study information was mailed to 399 men and women who underwent resection of a colon adenoma during a routine screening colonoscopy at Siteman Cancer Center. These individuals received a letter from their gastroenterologist inviting them to participate in the study. Individuals were then contacted by phone to confirm eligibility if interested. Eligibility criteria included individuals between the ages of 50 and 80 with no personal cancer history who were diagnosed with adenomatous polyp upon screening colonoscopy in previous 6 months and had no contraindications to beginning an exercise program, no previous diagnosis of diabetes, familial polyposis syndromes, ulcerative colitis or Crohn’s disease. Individuals were ineligible if they reported 30 or more minutes of moderate intensity physical activity 5 or more days per week or 20 or more minutes of vigorous intensity physical activity 3 or more days per week using the Behavioral Risk Factor Surveillance System physical activity questionnaire. Participants who were regular NSAID users also were excluded because the study included an exploratory aim examining change in serum inflammatory markers. The Washington University Institutional Review Board approved the study and written consent was obtained from all participants.

### Randomization

The allocation sequence was determined by random.org prior to participant enrollment. Sequentially numbered envelopes concealed the randomization to either group. The randomization was sealed in an envelope by a blinded staff member. The intervention coach opened the envelope following baseline assessment and informed participants. A blinded staff member completed all follow up data collection.

### Intervention

The SDCC Pilot was a group-based intervention weekly for four weeks followed by eight weeks of once-weekly phone-based follow-up. Outside of group meeting times, which included a group walk, participants were expected to walk on their own, progressing over time to reach their respective study arm goal. In previous versions, FSP has recommended a standard dose of 30 minutes of walking and 10,000 steps per day. This is consistent with the current federal physical activity guidelines for health [7]. Research in colon cancer survivors suggests that 60 minutes of physical activity is needed to prevent recurrence and improve survival [12], [13] as well as to manage weight [37], which is independently associated with colon cancer risk [38]. Thus, we added a study arm that delivered a higher dose of physical activity (60 minutes of walking, 13,000 steps per day).

The group sessions lasted four weeks and were led by intervention coach. The sessions consisted of individual progress reports, a brief walk of increasing duration, strategy discussion, and individual goal setting for the following week. Between sessions, participants walked on their own, wearing pedometers to self-monitor their daily steps. Participants logged their goals and daily step counts to monitor their progress. Coaching sessions were dedicated to different behavioral strategies each week. In week 1, participants learned about step counting basics and goal setting using the SMART (specific, measureable, attainable, realistic, time-limited) system. In week 2, participants discuss barriers to activity and the challenges of making trade-offs focusing on the benefits of physical activity and the sacrifices that may come with achieving their goal. During week 3, participants set new goals and work to brainstorm strategies that can help them achieve their step goal. In week 4, participants refine their goals and talk about relapse prevention. The intervention was targeted to the participant population by including discussion of the role of physical activity in colon cancer etiology.

The SDCC 10,000 step goal is readily achievable for individuals who are attaining already 6–8,000 steps/day in their usual daily activity and are adding 30 (2–3,000 steps) minutes of additional purposeful walking. However, data in chronically diseased and sedentary populations indicates daily step counts are likely to be much lower (3–5,000 steps/day) [26]. For those individuals whose baseline step count is lower and in the higher dose arm, the coach worked to progress them to a safe and reasonable goal driven by the time goal (30 vs 60 minutes/da of walking) and using their pedometer recorded step counts as a motivational and self-monitoring tool. To maintain attendance rates, participants who missed a session received follow-up calls and reminders for future sessions. In phase two, participants received eight weeks of brief phone support. Participants walked on their own, monitoring progress using pedometers. Participants were contacted by phone and asked to report their pedometer wear time, last daily step count and were given the opportunity to ask questions or get additional feedback as needed from the coordinator.

### Measures

Data collection was completed at Washington University School of Medicine. Baseline measures were taken prior to the initiation of the intervention and follow-up measures following the last week of intervention. To measure the outcome of step count change, independent of self-monitoring, participants wore a sealed blinded pedometer for one week at baseline and at follow-up and logged their wear time. Participants had to report wearing the pedometer for at least eight hours/day to be considered a valid day of wear. Only valid wear days were included and the average steps/day during the week was based only on days worn. We used the Omron HJ 720 IT pedometer (Omron HealthCare, IL), which has been shown to have higher validity in overweight and obese individuals. Wear time was estimated based on the hourly counts provided by the device output, which records counts per hour.

Participants also wore an Actigraph GT1M (Actigraph, Pensacola, FL) accelerometer for one week at baseline and at follow up to measure moderate/vigorous physical activity as an additional outcome. The accelerometer had to be worn a minimum of 10 hours to be considered a valid day. Data was collected in 60 second epochs. Data was processed using the ActiLife software (Actigraph, Pensacola, FL) and the Freedson equation was used to define activity intensity cutpoints and time in moderate/vigorous physical activity.

All study participants received $50 each for completing the baseline and follow-up assessments. Participants were also given an unblinded pedometer at the study conclusion.

### Analysis

To be conservative in estimating the intervention effect, in all analyses, participants who recorded baseline data, but did not record follow-up data were included in the analyses; with the baseline value carried forward as no change. Fisher’s exact tests were used to compare demographics between the two study arms because of small numbers in some cells. Paired t-tests were used to examine change from baseline to follow-up. T-tests were used to compare the intervention arms using an intent-to-treat analysis.

## Results

Recruitment began in June 2009 and enrollment was completed in December 2009. 265 of the 399 (66%) individuals sent an invitation letter were reached and screened via telephone. 101 did not meet inclusion criteria (38%) and 136 declined participation (51%). 28 qualified for the intervention, and 17 consented and enrolled. 16 were randomly assigned to one of two arms. One participant was unable to commit to study requirements after completing consent and baseline measures and was not randomized. 13 of the 16 enrolled (81%) completed the intervention (five in the standard dose arm and eight in the high dose arm) and12 (75%) completed the accelerometer protocol (Consort Diagram S1). The most common reasons for not qualifying were age, disabled/unable to walk, and already exercising regularly. Of the 3 who did not complete the intervention, 1 was unable to commit to the study requirements and 2 were lost to follow up. Of the 16 participants, 12 recorded 5 or more days of valid accelerometer wear time, and 4 failed to meet the accelerometer wear time requirements at baseline. Of the 13 participants who completed the intervention, 12 recorded 5 or more days of valid accelerometer wear time one recorded 4 days. Pedometer wear time was similar, with 11 participants recording at least 5 days of valid wear time, 2 participants recording 4 days, 2 participants had 3 days and 1 had 2 days of valid wear time. At follow-up, 11 of the 13 participants recorded 5 or more valid days of pedometer wear time, with the remaining 2 recording 1 day and 3 days, respectively.

The population ranged in age from 45–66. 63% (n = 10) of the participants were African American (Table S1) and most (81%) were female. The participants were largely employed (63%) and most had some post-high school education. There were no significant differences between the two intervention arms on any sociodemographic factors.

Participants recorded steps typical of chronically diseased adults, with a mean of 4549 (standard deviation(sd) 2720) steps/day at baseline (Table S2). Participants were also insufficiently physically active as measured by accelerometer, recording 96 (sd 106) minutes/week of moderate/vigorous intensity physical activity. The intervention significantly increased mean physical activity levels, measured by blinded pedometer (1791 (sd 2065) steps/day) and accelerometer (105 (sd117) minutes moderate/vigorous intensity physical activity/week). Participants in the standard dose arm increased moderate/vigorous activity by 65 (sd130) minutes/week and increased steps by 836 (sd 1284) per day. In the high dose arm, participants increased steps by 2746 (sd 2325) per day and moderate/vigorous physical activity by 133 (sd107) minutes/week. Both arms failed to reach the intervention daily step target, recording an average of 6340 (sd 3363) steps/day at follow-up. However, this was a significant change from baseline (p = 0.003). Despite not meeting the step count target at follow-up, participants in both arms met the time-based exercise prescription, recording a mean of over 200 minutes/week of moderate/vigorous physical activity and qualifying them as meeting the US Physical Activity Guidelines. This was also a significant increase over baseline (p = 0.01).

There was no significant difference between the arms in steps per day or minutes of moderate/vigorous physical activity per week at baseline. We found an increase in physical activity in both arms as measured by accelerometer and blinded pedometer. The difference between arms was not significant for accelerometer measured physical activity (p = 0.34) and was marginally not significant for steps (p = 0.07).

## Discussion

Our pilot intervention yielded a significant increase in physical activity as measured by two different objective measures- accelerometer and blinded pedometer. Despite participants failing to achieve the stated step count intervention target at follow-up for either dose arm, participants did meet the intervention time-based target dose in both arms. While the high (60 min/day) dose arm had a target daily physical activity level that was twice current physical activity recommendation, the higher dose arm did not achieve a significantly higher level of physical activity than the standard (30 min/day) dose arm.

Despite walking being the most commonly reported physical activity [39], [40], measuring walking presents numerous challenges, including biased recall of occurrences, speed, and/or intensity [41]–[43]. Thus, objective measurement tools that can detect gradations in walking behavior are useful physical activity measures. The step counts recorded by our population (mean 4549/day at baseline) were comparable to previous reports of sedentary populations [26], [27].

Pedometers are effective physical activity promotion tools as they can provide immediate feedback in the form of step counts, thereby facilitating individual-level behavior modification [44]–[47]. For example, a study by Croteau found that a minimal contact, self-managed, pedometer-based intervention resulted in a significant increase in the average daily steps of participants from 8565 (+/−3121) to 10538 (+/−3681) at follow-up in 37 men and women, a change of 1973 steps [45]. The impact of our intervention was similar, though we noted that the change was larger among those given a larger intervention target dose.

Despite not achieving the intervention step count target, both arms recorded more than 150 minutes of moderate/vigorous physical activity per week on the accelerometer at follow-up and exceeded the intervention target dose for duration. This may suggest that pedometer wear time was not complete during the assessment periods, as the accelerometer physical activity estimates employ an algorithm that accounts for daily wear time, or that the pedometers underestimated the step count [48]. Our study was also subject to other limitations, including our small sample size. While the small sample size was part of the pilot study process, it may have reduced our ability to detect differences in the intervention arms.

Our response rate provides useful information for planning recruitment to physical activity promotion programs in populations at increased risk for colon cancer. The response rate was similar to other “cold contact” approaches to health behavior research [49]. The study also has several strengths including our reliance on an evidence-based intervention and objective assessment of physical activity change.

This pilot intervention provided important information that can be used in larger trials. This 12 week physical activity intervention of minimal supervision can result in significant increases in physical activity, sufficient to meet the US Physical Activity Guidelines [7]. While pedometers are useful tools for self-monitoring and physical activity promotion, their display interface may induce reactivity such that they may not accurately reflect changes in behavior during an intervention. We attempted to minimize this by blinding the pedometer used for assessment purposes, but participants also wore their intervention pedometer at follow-up. Importantly, despite initial concerns that a target of 60 minutes/day would be too aggressive or difficult for this largely sedentary population to achieve, both study arms increased physical activity by more than twice the intervention recommendation at follow-up. Some of this time likely reflects usual daily activity, given the physical activity level recorded at baseline, but the increase from baseline to follow-up was significant.

Our pilot study indicates that a minimal contact intervention can generate significant changes in physical activity among individuals at elevated risk for colon cancer. However, a more contact-intensive intervention may be necessary to achieve the target intervention dose and this should be evaluated in future studies. Furthermore, because the study did not achieve the target dose, the potential impact of the intervention on colon cancer risk is not clear. Future studies should also evaluate whether this minimal contact intervention results in changes in endpoints more closely tied to colon cancer risk.

## Supporting Information

Table S1 **Step Down Colon Cancer Pilot Participants’ Baseline Characteristics.**
(DOCX)

Table S2 **Step Down Colon Cancer Pilot Objective Physical Activity.**
(DOCX)

Consort Diagram S1 **Step Down Colon Cancer CONSORT Diagram.**
(DOCX)

Checklist S1 **CONSORT 2010 Checklist.**
(DOC)

Protocol S1 **Trial Protocol.**
(DOCX)
