# Supplementary material for: Promoting Physical Activity in Patients with Colon Adenomas: A Randomized Pilot Intervention Trial
Source: PLoS One. 2012 Jul 13;7(7):e39719. doi: 10.1371/journal.pone.0039719 (PMC3396639; doi:10.1371/journal.pone.0039719)
Supplement: Table S1 — Step Down Colon Cancer Pilot Participants’ Baseline Characteristics. (DOCX) [file pone.0039719.s001.docx]

Table S1. Step Down Colon Cancer Pilot Participants’ Baseline Characteristics

| **Characteristic** | **30 minute dose (n=8)** | **60 minute dose (n=8)** | **p-value** |
| --- | --- | --- | --- |
| Race |  |  | 1.0 |
| Black (%) | 63 | 63 |  |
| White (%) | 37 | 37 |  |
| Employed (%) | 63 | 63 | 1.0 |
| Married (%) | 63 | 37 | 0.62 |
| Female (%) | 63 | 100 | 0.20 |
| Education |  |  | 1.0 |
| High school/GED (%) | 12 | 6 |  |
| Some college/ associates degree/ vocational training (%) | 63 | 63 |  |
| College or graduate school (%) | 25 | 25 |  |
| Age |  |  | 1.0 |
| <50 (%) | 0 | 13 |  |
| 50-<60 (%) | 50 | 37 |  |
| 60+ (%) | 50 | 50 |  |
