# Supplementary material for: Promoting Physical Activity in Patients with Colon Adenomas: A Randomized Pilot Intervention Trial
Source: PLoS One. 2012 Jul 13;7(7):e39719. doi: 10.1371/journal.pone.0039719 (PMC3396639; doi:10.1371/journal.pone.0039719)
Supplement: Table S2 — Step Down Colon Cancer Pilot Objective Physical Activity. (DOCX) [file pone.0039719.s002.docx]

Table S2. Step Down Colon Cancer Pilot Objective Physical Activity

* Baseline vs follow-up, p<0.05. No other differences statistically significant.

|  | Baseline | Follow-up (12 weeks) | Change |
| --- | --- | --- | --- |
| **Number of Steps (mean (SD) per day)**  **-Blinded Pedometer (n=16)** | **4549 (2720)** | **6340 (3363)** | **1791 (2065)*** |
| 30 min group | 4991 (3453) | 5827 (3714) | 836 (1284) |
| 60 min group | 4108 (1866) | 6854 (3137) | 2746 (2325) |
| **Minutes of Moderate/Vigorous Activity (mean per week)**  **-Accelerometer (n=12)** | **96 (106)** | **201 (208)** | **105 (117)*** |
| 30 min group | 100 (159) | 164 (289) | 65 (130) |
| 60 min group | 93 (60) | 226 (147) | 133 (107) |
