# Supplementary material for: Promoting Physical Activity in Patients with Colon Adenomas: A Randomized Pilot Intervention Trial
Source: PLoS One. 2012 Jul 13;7(7):e39719. doi: 10.1371/journal.pone.0039719 (PMC3396639; doi:10.1371/journal.pone.0039719)
Supplement: Consort Diagram S1 — Step Down Colon Cancer CONSORT Diagram. (DOCX) [file pone.0039719.s003.docx]

**Step Down Colon Cancer CONSORT Diagram**

Recruitment packets mailed to colonoscopy recipients with polyp. (n= 399)

Letters returned

(n= 2)

Assessed for eligibility

(n=265)

Could not be reached

(n= 132)

Excluded (n= 237)

- Not meeting inclusion criteria

(n= 101)

- Refused to participate

(n= 136)

*

Allocated to intervention (n= 8 )

- Received allocated intervention (n= 7)
- Did not receive allocated intervention (Unable to meet study requirements) (n= 1)

Analyzed (n= 8)

Excluded from analysis (n= 0)

Completed follow-up (n= 8 )

Discontinued intervention

(n= 0)

Allocated to intervention (n= 8 )

- Received allocated intervention (n= 8)
- Did not receive allocated intervention (n= 0)

**60 Minute Walking Group**

**30 Minute Walking Group**

## Follow-Up

## Analysis

Completed follow-up (n= 5)

Discontinued intervention

(No show, unable to reach)

(n=2)

#

Qualified (n= 28)

Consented (n=17)

## Allocation

Analyzed (n= 5)

Excluded from analysis (n= 0)
