# Supplementary material for: Promoting Physical Activity in Patients with Colon Adenomas: A Randomized Pilot Intervention Trial
Source: PLoS One. 2012 Jul 13;7(7):e39719. doi: 10.1371/journal.pone.0039719 (PMC3396639; doi:10.1371/journal.pone.0039719)
Supplement: Protocol S1 — Trial Protocol. (DOCX) [file pone.0039719.s005.docx]

**Step Down Colon Cancer Risk:**

A Pilot Intervention for Colon Cancer Risk Reduction

PI: Kathleen Y. Wolin, ScD

**A. BACKGROUND AND SIGNIFICANCE**

Colon cancer is the third most commonly occurring cancer in men and women in the US.^1, 2^ Evidence linking physical activity with a reduced risk of colon cancer is consistent and convincing.^3^ Recent analyses suggest that nearly one third of cancer cases can be attributed to nutrition and physical activity related factors.^3^ Despite the benefits of physical activity, nearly 75% of the population fails to meet recommended physical activity levels.^4-6^

In this study, we will refine and pilot test an intervention to promote walking among individuals with a previous polyp. Our goal is to reduce risk of colon cancer by triggering changes in important biomarkers, including IL-6, PGE-2, insulin, and C-peptide through increased physical activity.

**A.1. Physical activity and colon cancer.** Observation studies consistently link physical activity with a reduced risk of colon cancer.^3, 7-20^ Despite the consistency of the evidence, many aspects of the relationship remain to be elucidated.

Previous studies have reported a reduced risk of colon cancer with engagement in moderate intensity activity.^13, 21^ Recent analyses in the Nurses’ Health Study, the largest prospective study to examine this phenomenon to date, found a significant risk reduction among women walking at least two hours per week.^22^ Given that walking is a commonly reported activity, and is the most common activity among middle-aged and older women^23, 24^, these results have important implications for prevention. The components from the biologic model of the hypothesized association between walking and colon cancer risk reduction are outlined in Figure 1.

**Figure 1. Physical activity and colon cancer**

Physical activity

Colon cancer

insulin

IL-6

PGE-2

C-peptide

+

+

+

+

+

**A.2. Biomarkers and colon cancer**

**A.2.a. Insulin and colon cancer.** Insulin is involved in cell proliferation and apoptosis. As a result, the role of insulin in carcinogenesis has been a focus of much research. Several lines of evidence support the hypothesis that hyperinsulinemia promotes the growth of colon tumors. Colon tissue has insulin receptors^25^ and insulin stimulates the growth of both normal and colon cancer cells in vitro.^26, 27^ Insulin-like growth factors may also promote colon tissue growth.^28^ The association seen between type II diabetes, which often results in hyperinsulinemia due to insulin resistance, and colon cancer provides additional support for the role of hyperinsulinemia in colon carcinogenesis.^29-32^ Animal studies also support the role of insulin in colonic carcinogenesis.^33^

Observational studies have found associations between insulin and both risk of colon cancer^34, 35^ and risk of colon polyps, precursors to colon tumors.^36-38^ Individuals in the highest quartile for insulin had a twofold increased risk for colon cancer compared to those in the lowest quartile.^35^

C-peptide is an insulin production by-product created when proinsulin is cleaved into insulin and C-peptide. C-peptide is more stable and, therefore, a better marker of insulin secretion; levels reflect how much insulin the body is producing. Recent research^39-42^ has found a positive association between C-peptide levels and risk of colon cancer as well as risk of colorectal adenoma,^43^ further supporting the hypothesis that hyperinsulinemia may play an important role in colon carcinogenesis. Thus, it has been suggested that targeting hyperinsulinemia may be an effective colon cancer prevention strategy.

**A.2.b. Inflammation and colon cancer.** Several lines of research suggest a role for inflammation in colon carcinogenesis. Non-steroidal anti-inflammatory drugs (NSAIDs) have shown promise as chemopreventive agents, but concerns exist regarding the side effects associated with long term use.^44^ COX-2 specific inhibitors have shown protection against colon polyps, but have been found to have adverse cardiovascular effects.^44-46^ The mechanism behind the protective effect of NSAID use is hypothesized to be in part due to the inhibition of COX-derived prostaglandin E2 (PGE-2), which promotes tumor carcinogenesis.^47^

Prostaglandin levels are increased in COX-2 mediated inflammation (PGE-2 is a COX-2 metabolite). PGE-2 is typically released by blood vessel walls in response to infection or inflammation. PGE-2 is the most abundant prostaglandin in colorectal cancer^48^ and promotes tumor progression by stimulating cell proliferation and angiogenesis, inhibiting apoptosis and modulating immunosuppression.^49^ Thus, factors which reduce levels of PGE-2 are also hypothesized to reduce colon cancer risk.

Research has also demonstrated a key role for interleukin-6 (IL-6) in the inflammation – colon cancer relationship. IL-6 is a pro-inflammatory cytokine secreted by T-cells and macrophages. IL-6 promotes the transition from acute to chronic inflammation.^50^ Emerging research indicates a predominant role for the IL-6 pathway in bowel inflammation.^51^ IL-6 associated genetic polymorphisms have shown an increased risk of colon cancer.^52^ Observational research has provided preliminary support for this model, demonstrating a positive association between serum IL-6 levels and colon cancer.^53^ IL-6 levels were also correlated with larger tumor size and metastasis and levels increased in a stage-related manner. Finally, there is some indication that IL-6 may be related to insulin resistance in cancer patients.^54^

**A.3. Physical activity and biologic markers.**

**A.3.a Physical activity (PA) and insulinemia.** PA increases insulin sensitivity ^55-60^ with the effects of a single exercise bout potentially lasting for several days,^61, 62^ resulting in decreased plasma insulin levels.^60, 63-71^ There is also evidence that walking, in particular, decreases insulin^72, 73^ and increases insulin sensitivity.^74, 75^ Physical activity is also inversely associated with C-peptide levels.^40, 66, 76-80^ However, some research suggests that vigorous physical activity may be necessary to reduce C-peptide levels.^76, 78^ The associations seen for vigorous physical activity may reflect that more intense physical activities are easier to recall.^81^ Most observational data is from cross sectional studies. Data on the effect of sustained changes in physical activity on C-peptide levels is limited.

**A.3.b. Physical activity and inflammation.** Interest in the association between physical activity and inflammation has traditionally been limited to cardiovascular research where the inflammatory factors of interest include c-reactive protein (CRP), white blood cell count (WBC) and fibrinogen.^82^ Population-based cross-sectional studies have generally found an inverse relation between physical activity and CRP level with similar associations for WBC and fibrinogen. ^82-84^

Inverse associations between self-reported physical activity and levels of other inflammatory markers have been found.^85^ In cross-sectional analyses, physical activity was inversely associated with IL-6.^68, 86^ In addition, a significant decrease in IL-6 was found following a 12-week aerobic training intervention in 24 men^87^ and a separate 10-week intervention in 20 men and women.^88^ In a 10 month study of older adults, Kohut and colleagues found that thrice weekly aerobic training, but not thrice weekly flexibility training, reduced IL-6 levels in 87 men and women.^89^

Less data is available on the association between physical activity and PGE-2 but given the hypothesized role in inflammatory-related colon cancer pathways noted above and the association broadly seen for physical activity and inflammation, it merits further investigation. In addition, while IL-6 is produced by visceral adipocytes, PGE-2 represents a mechanistic pathway independent of weight.

Based on the above studies, we will measure PGE-2, insulin, C-peptide and IL-6.

**A.4. Interventions to prevent colon cancer.** Numerous randomized controlled trials of lifestyle risk factors for colon cancer prevention have been conducted in recent years, but all have focused on dietary changes or taking supplements with one exception.^45, 90-92^ That study randomized participants to a 12 month moderate to vigorous aerobic exercise program or delayed two-month intervention and examined changes in colon cell architecture and proliferation.^93, 94^ Given the wealth of observational data demonstrating the large risk reductions that can occur with participation in physical activity, it is surprising that more randomized controlled trials of colon cancer prevention have not been conducted.

There is a lack of interdisciplinary research that combines theoretically driven behavior change interventions and etiologically relevant endpoints in a rigorous randomized controlled trial design for colon cancer prevention or risk reduction. This study improves on previous work by measuring PA and biomarkers. The lack of focus in the existing literature on physiologic parameters in physical activity studies, and the lack of quality interdisciplinary research, is a weakness of the existing literature. Our study is innovative and important in its ability address these multiple levels of outcomes.

**A.5.Measuring physical activity.** Despite walking being the most commonly reported physical activity,^23, 24^ recall of walking presents numerous challenges, including recall of occurrences, speed, and/or intensity.^95-97^ Thus, objective measurement tools that can detect gradations in walking behavior are useful physical activity measures.

Pedometers are easy to use, relatively low cost, reliable and accurate.^98^ Pedometers capture the vertical accelerations associated with normal ambulation and thus, measure the total number of steps taken over a given period of time (i.e. day, week). Most studies suggest that pedometers have acceptable validity when compared to more comprehensive objective measurement devices like accelerometers, particularly when measurement is focused on walking behaviors.^99, 100^

The combination of the high frequency of walking as a physical activity and their comparatively low cost makes pedometers well suited for population-based research. In addition, pedometers are non-invasive, low burden tools that are socially acceptable and practical in-vivo measures of physical activity.

**A.5.a. Average Daily Pedometer Step Counts:** A review of 32 observational and intervention studies suggests that daily step counts range from: (1) 7-13,000 steps per day for healthy younger adults; (2) 6-8,500 steps per day for healthy older adults; and (3) 3,500-5,000 steps per day for sedentary individuals and those with disabilities of chronic illness.^101^ Recent research has attempted to derive a step count recommendation associated with a 30-minute interval of activity (the current American College of Sports Medicine (ACSM) physical activity recommendation for moderate intensity physical activity^5^). These studies have yielded varying results, but have suggested step counts in the range of 3000- 4000 steps are accumulated during 30-minutes of walking.^102, 103^

**A.5.b. Use of Pedometers as Health Promotion Tools:** Evidence is emerging that pedometers, in particular, are effective physical activity promotion tools as they can provide immediate feedback in the form of step counts, thereby facilitating individual-level behavior modification.^102, 104-106^ For example, a study by Croteau found that a minimal contact, self-managed, pedometer-based intervention resulted in a significant increase in the average daily steps of participants from 8565 (+/- 3121) to 10538 (+/- 3681) at follow-up in 37 men and women.^105^

**A.5.c. The 10,000 Steps Per Day Goal:** For pedometers to be successfully implemented in physical activity promotion interventions, they must be paired with clear and empirically-meaningful step count recommendations. The 10,000 steps per day recommendation has become increasingly popular with the public, media, and interested corporations (including recent marketing efforts by McDonald’s and Kellogg cereals).^107^ Existing pedometer-based physical activity promotion studies have also adopted this intervention message. While there has been empirical support for the 10,000 steps per day goal in clinical and monitored populations,^101, 108, 109^ the goal may initially be too ambitious for sedentary and older adult populations, who may take as few as 3,500 – 5,500 steps per day.^101^ A 10,000 steps per day goal for these individuals then, amounts to a two- to three-fold increase in daily physical activity – a recommendation that is very likely to promote attrition.^110^ Given that there is some evidence to suggest that a single 10,000 steps per day goal may in fact be an ineffective physical activity promotion strategy, particularly among the largely sedentary populations who could benefit most from increases in their physical activity patterns, interventions that employ stepwise physical activity goals and incorporate other ways of promoting physical activity in addition to step count recommendations may be most effective.

1. **SPECIFIC AIMS**

To design and evaluate the feasibility of a pilot version of a colon cancer prevention intervention focused on walking. Individuals (n=20) with a polyp found on screening colonoscopy in the last six months (i.e., a colonoscopy to detect polyps or adenocarcinoma) will be enrolled and randomized to an evidence-based intervention recommending one of two physical activity recommendations (30 or 60 minutes). Participants in both conditions will receive the First Step intervention, which has previously been tested and found to be efficacious for increasing physical activity in clinical populations. First Step recommends walking most days of the week using a daily step count goal to monitor and motivate behavior change.^111, 112^ Outcomes will be measured at one month and three months post baseline. We anticipate larger changes in the primary outcomes in the Plus group than the standard intervention group.

Primary Outcomes: Blood levels of insulin, C-peptide, IL-6, and PGE-2

Secondary Outcome: Change in physical activity level as measured by blinded pedometer.

The specific aims are:

**Primary Aim:** To conduct a dose response pilot trial of low (30 min/day) or high (60 min/day) dose exercise in men and women at increased risk of colon cancer. The major outcomes are changes in serum levels of four risk-related biomarkers: insulin, C-peptide, IL-6 and PGE-2.

Hypothesis 1: Exercise will decrease serum markers in a dose response manner.

**Secondary Aim.** To compare changes in the secondary outcome of physical activity over three months.

Hypothesis 2: Participants in the 60 minute intervention will have significantly higher physical activity levels than those in the 30 minute intervention at three months.

1. **RESEARCH DESIGN AND METHODS**

**C.1. Overview of research design and study.** The overall objective of this study is to compare two doses of physical activity delivered through existing walking intervention paradigm in 20 individuals at increased risk for colon cancer and evaluate the dose of activity necessary to change colon cancer risk related serum biomarkers. This 12-week two-arm randomized controlled trial will administer the First Plus walking program prescribing two different doses (30 minutes vs 60 minutes) of physical activity.^111, 112^ This study builds on previous physical activity interventions with cancer outcomes where 12 week interventions are common.^113-118^ The subjects in the study will be recruited from the gastroenterology practice at Barnes Jewish Hospital / Washington University School of Medicine (BJH/WUSM). The gastroenterology practice sees approximately 4000 patients per year for colonoscopy. Of these, half present for screening or surveillance procedures and approximately 20% of these present with adenomatous polyps. The patients are representative of the larger patient population at BJH/WUSM, with half being female and one third African American. Children are not screened for colon cancer and are thus not included in this study. The study will be coordinated and intervention delivered by Cindi Inman, MS, RD, LD and overseen by Dr. Wolin. Ms. Inman has a master’s degree in exercise physiology and extensive experience managing health promotion research studies. Angela Tanner will assist with data collection and management under Dr. Wolin’s supervision. A study schema (Figure 2) is provided below.

Eligibility screening

Blinded pedometer protocol & accelerometer protocol (n=20)

Baseline assessment

- Fasting blood draw (insulin, C-peptide, IL-6, PGE-2)
- Height and weight
- PPAQ
- Social support
- Self efficacy
- Safety
- IPAPS
- HINTS benefits of physical activity
- Social norms
- Sociodemographics (age, race/ethnicity, gender, education, occupation, income)
- Cancer risk factors (family history, tobacco, hormones, diet)

Phase I assessment:

Baseline assessment minus sociodemographic and cancer risk factor questions

**PLUS**

Pedometer & accelerometer data download

Phase I assessment:

Baseline assessment minus sociodemographic and cancer risk factor questions

**PLUS**

Pedometer & accelerometer data download

randomization

60 minute dose

(n=10)

30 minute dose

(n=10)

Week 12

Week 5

Pedometer data download

**Figure 2. STUDY SCHEMA**

Week 10

**C.2. Recruitment**

**C.2.a. Screening procedures.** The first 20 interested and eligible patients will be enrolled. To identify patients, staff in the BJH/WUSM gastroenterology practice have agreed to review practice records to identify individuals meeting eligibility criteria (see below). These individuals will be sent a letter from their gastroenterologist inviting them to participate in Dr. Wolin’s study. Individuals may contact study staff to opt out of the study. Individuals who do not contact study staff to opt out will be contacted by the Recruitment Enhancement Core (REC) of the ICTS to invite them to participate and determine eligibility.

REC staff will screen interested individuals for a history of cancer, diagnosis of familial polyposis syndromes, ulcerative colitis, Crohn’s Disease and medical indications that counter-indicate exercise. Participants will answer a brief questionnaire (based on the BRFSS) to determine current physical activity levels and participants’ current use of NSAIDs. Staff will explain the overall study design to eligible individuals. Specifically, potential participants will be told that the study is a randomized behavior change intervention for colon cancer recurrence where that participants will be assigned by chance to one of two physical activity interventions. Staff will detail the length of the study and that participants may be required to do any or all of the following: (1) attend a thirty- to sixty-minute behavior change sessions at the BJH/WUSM once a week for a month, (2) answer weekly telephone-based questionnaires, (3) complete evaluations at BJH/WUSM every four to for three months, including an evaluation at the study end.

Participants will be made aware that by enrolling in the study they may be subject to an in person health evaluation and that all participants must return to BJH/WUSM in three months to complete additional assessments.

**C.2.b. Eligibility.** Eligibility is based on the following criteria: (1) aged 50 to 80; (2) no personal cancer history; (3) found to have an adenomatous polyp upon colonoscopy at BJH/WUSM in the previous six months; (4) no contraindications to beginning an exercise program; (5) no previous diagnosis of familial polyposis syndromes; (6) no previous diagnosis of ulcerative colitis or Crohn’s disease;

**Additional Exclusion Criterion: NSAID use.** Participants who are regular NSAID users will be excluded as this may interfere with the measurement of inflammatory marker outcomes. Regular use is defined as taking 80mg or more per day of aspirin, ibuprofen, naproxen or other NSAID 5 or more days of the week.

**Additional Inclusion Criterion: Sedentary.** Individuals who report 30 or more minutes of moderate intensity physical activity five or more days per week or 20 or more minutes of vigorous intensity physical activity three or more days per week will be considered physically active and thus, ineligible. Participants will be administered the Behavioral Risk Factor Surveillance System (BRFSS) physical activity questionnaire as it is a brief screening tool useful for this purpose. This instrument asks participants to report on physical activity of at least moderate intensity performed in bouts of at least 10 minutes during the previous week. Individuals who report having been diagnosed with Type 1 or Type II diabetes will be included.

**C.3. Measures**

**C.3.a. Objective physical activity measurement.** Within two weeks of their baseline assessment all participants will come in to complete the consent and be fitted with a blinded pedometer with instructions on placement and wear. Participants will be instructed to wear the device for the 7 days prior to their baseline visit. All pedometers will be blinded to minimize behavior change in response to the assessment. Logs will be collected at the baseline visit. Log data will be used to determine days and hours of wear. At the baseline visit, pedometers will be collected and data will be downloaded to the study computer and processed using manufacturer software. Participants will also be provided with an accelerometer during this period for assessment of total physical activity levels. Participants will also provide objective physical activity data at follow-up as outlined below.

**C.3.a.1 Pedometer.** We will use the Omron HJ 720IT pedometer in this study. This pedometer displays seven days of step count data (when not blinded and sealed), but can store up to 41 days of step count data in the memory for later download. Research has suggested that the Omron pedometer technology may have superior accuracy among elderly and obese individuals.^119, 120^ Given the high prevalence of obesity in the general population^121^ and the age range of our eligibility criteria, we believe it is prudent to use the Omron device. Participants will complete logs and record daily hours of wear.

**C.3.a.2. Accelerometer.** We will use the Actigraph brand accelerometer in this study. The accelerometer will allow us to estimate the participants’ total physical activity level including minutes of moderate or vigorous intensity physical activity as well as a summary indicator of physical activity that accounts for both intensity and duration, expressed as metabolic equivalent (MET) minutes per week. Finally, it will allow us to estimate the participants’ energy expenditure.

Accelerometers are generally recognized as a “gold-standard” approach for the objective measurement of physical activity. Accelerometers record the g-forces common to movement and translate them into data that reflect the frequency and intensity of movement, sampled at set intervals. With accelerometry the duration time spent at defined intensities of activity can be determined, which allows for the more valid assessment of physical activity. In interventions of physical activity behavior change, accelerometers provide the most valid estimates of change in energy expenditure.

**C.3.b. Physiologic assessment.** At the baseline and follow-up visit, all participants will undergo a fasting blood draw. Staff will also measure participants’ weight and height.

**C.3.b.1. Serum marker measurement.** Blood will be collected after a 12 hour fast. Participants will also be instructed not to consume alcohol for 18 hours prior to the blood draw or take NSAIDs for 24 hours prior to blood draw. Because daily physical activity is part of the intervention message, participants will not be asked to terminate physical activity, but limit it. Specifically, participants will be asked not to participate in vigorous physical activity for 12 hours prior to the blood draw and not to participate in any moderate or vigorous intensity physical activity for three hours prior to the blood draw.

The Center for Clinical Studies (CCS) will oversee the physiologic assessment, including sample collection, processing, and storage. Samples will be processed in order to allow subsequent analysis of levels of insulin, C-peptide, IL-6 and PGE-2. Blood will be processed and stored as serum, EDTA plasma, and red blood cells. Each participant’s baseline and follow-up blood samples will be assayed in the same batch. In addition, an equal number of samples from each study arm will be included in each batch.

**C.3.b.2. Body composition assessment.** Body weight and height will be measured in duplicate using a digital scale and tape measure constructed specifically for research quality measures. We will also measure waist and hip circumference.

**C.3.c. Questionnaire.** Following the physiologic assessment and pedometer return, participants will be provided a questionnaire that includes physical activity, potential confounder and sociodemographic information. Measures are summarized in Table 2 and are described in detail below.

**C.3.c.1. Self-reported physical activity.** As a supplement to the step count data, participants will complete a physical activity questionnaire. We will use the Paffenbarger Physical Activity Questionnaire (PPAQ) which assesses moderate and vigorous intensity physical activity with distinct items to measure walking.^95, 122^ The PPAQ also allows measurement of both leisure time only and total (leisure time plus occupational and transport) physical activity. This validated instrument was selected because it provides an assessment of total physical activity while also employing several detailed walking items. The information in the PPAQ supplements that provided by the accelerometer and pedometer by providing information about context/domain of physical activity.

**C.3.c.2. Modifying mechanisms.** We will also collect information related to the intervention constructs using validated published scales whenever possible. Self-efficacy for physical activity will be measured using the Exercise Confidence Survey.^123^ Social support for physical activity will be measured using the Sallis Social Support Scale.^124^

| **Table 2. Survey measures** | | |  |  |
| --- | --- | --- | --- | --- |
| **Construct** | **Measure** | **Baseline** | | **Follow up** |
| Self-reported physical activity | Paffenbarger Questionnaire | X | | x |
| Sociodemographic | Age | X | |  |
|  | Marital Status | x | |  |
|  | Race/ethnicity | X | |  |
|  | Gender | X | |  |
|  | Education | X | |  |
|  | Income | X | |  |
|  | Occupation | X | |  |
|  | Immigrant status | X | |  |
| Other cancer risk factors | Family history of colorectal cancer | X | | x |
|  | Current and past tobacco use | X | | x |
|  | Hormone use (women only) | X | | x |
|  | Diet (Willett Questionnaire) | X | | x |
| Self efficacy | Exercise Confidence Survey | X | | x |
| Social support | Sallis Social Support Scale | X | | x |
| Neighborhood safety | Day/Night; Home/work | X | | x |
| Benefits of physical activity | HINTS questions | X | | x |
| Neighborhood walkability and access | IPAPS self administered module | X | | X |
| Length of residence |  | X | |  |
| Perceived neighborhood disorder |  | X | | X |
| Depression | CES-D | X | | X |
| Perceived Stress |  | X | | X |
| Coping strategies | Brief COPE | X | | X |
| Urban life stress | Urban Life Stress Scale | X | | X |

**C.3.c.3. Other factors potentially related to physical activity**. We will measure the perceived benefits of physical activity using the HINTS survey items.^125^ This instrument asks participants: (1) As far as you know, does physical activity or exercise increase the chances of getting some types of cancer, decrease the chances of getting some types of cancer, or does it not make much difference? (2) Can exercise help to lower the chances of getting some types of cancer or does exercise not make much difference? We will also administer the International Physical Activity Prevalence Study environmental module which queries participants about their neighborhood and its walkability as well as access to physical activity and recreation facilities. We will assess perceived neighborhood safety by asking participants, “how safe is the neighborhood you live in during the daytime?” We will also ask about the safety of the neighborhood they live in at night and the neighborhood they work in. Response options will be “safe”, “a little unsafe” and “not at all safe”. In previous work by the research team, these questions were associated with daily step count.^126^ Additional items on stress ^127-131^, neighborhood environment ^132, 133^, depression^134^, and coping mechanisms^135^ will also be included on the questionnaire.

**C.3.c.3. Other measures.** The baseline and follow-up questionnaires will also query sociodemographic variables (age, race/ethnicity, gender, education, household income, current occupation), family history of colon cancer, current and past tobacco use, diet and supplement use (using the Willett food frequency questionnaire) and use of hormones (in women).

**C.4. Randomization.** Following the baseline assessment, study staff will assign participants to an intervention condition using a random numbers program. An equal number of individuals will be assigned to each condition (10 low dose, 10 high dose). Staff doing the randomization will not participate in the eligibility screening or baseline assessment and will not have access to information about participants.

**C.5. Intervention.** Following the baseline assessment, participants will be randomly assigned to one of two doses of physical activity delivered through the First Step program.

**C.5.a. First Step Program.** The First Step Program (also published as Manpo-Kei^111^) is a theoretically-based two phase intervention that aims to promote adoption of physical activity, specifically walking, and adherence to the newly adopted behavior.^112^ The program draws on social cognitive theory and the Transtheoretical Model of behavior change. The first phase lasts one month and is comprised of four weekly facilitated group meetings. The group meeting covers individual progress reports, brief group walk of increasing duration, group strategy discussion, and individual goal setting for the following week. Participants use pedometers and logs to monitor their progress. Between the weekly sessions, participants wear pedometers to monitor their daily steps. Participants log their goals and daily step counts. The program addresses self-efficacy, outcome expectations and social support in line with social cognitive theory and moves participants through the phases (precontemplation, contemplation, preparation, action and maintenance) of the Transtheoretical Model. In the second phase, lasting eight weeks, participants receive weekly phone contact. The First Step Program aims to progress participants to a steps per day goal. The First Step Program has successfully increased physical activity in several diabetic patient populations.^136, 137^ The First Step Program has resulted in significant changes in waist girth.^137, 138^ Studies have found mixed results for changes in weight.^137, 138^ Changes have not been seen for waist to hip ratio^137^, or insulin.^136^.

**C.5.b. Dose.** To date, the First Step Program has administered a single dose recommendation of 30 minutes of walking and 10,000 steps per day. This is in line with the current federal physical activity guidelines for health.^6^ Research in colon cancer survivors suggests that 60 minutes of physical activity is needed to prevent recurrence and improve survival. This is also the amount of activity indicated as necessary to maintain a healthy weight. Thus, we will compare the First Step Program as previously delivered in diabetics, with a modified version that delivers a higher dose of physical activity (60 minutes of walking, 13,000 steps per day).

**C.6. Intervention evaluation and outcome measurement**

**C.6.a. Objective physical activity.** One week prior to the conclusion of the study, participants will come into receive a blinded sealed pedometer to wear for seven days. Participants will again be given an Actigraph device to wear for seven days. At the end of the seven day period, all participants will return to Washington University for their study conclusion evaluation visit. Data from the devices will be processed as at the baseline visit. Participants will complete a questionnaire and undergo a final physiologic examination and blood draw.

**C.6.b. Physiologic measures.** Participants will undergo physiologic measurement identical to baseline at the end of the study. We will measure height and weight, as well as insulin, C-peptide, IL-6 and PGE-2 levels. Blood will be processed and stored as serum, EDTA plasma, and red blood cells.

**C.6.c. Questionnaire.** Following the physiologic assessment and pedometer return, participants will be provided a questionnaire identical to that used at baseline that includes items querying physical activity, potential confounder and sociodemographic information.

**C.7. Incentives.** All study participants will receive $50 each for completing the baseline assessment as well as $50 for attending the 3 month (study end) visit. Participants will also be allowed to keep their study pedometers.

**C.8. Retention strategies.** To ensure high retention rates of study participants, we will employ several strategies including providing detailed information on risks associated with study participation to build trust, accessing participant information through their physician to help reinforce the clinical support for the study program, and the use of incentives. In addition, at enrollment we will collect multiple sources of contact information from participants including home, work and mobile phone numbers as well as email addresses where available. We will also request the name and contact information for a friend or family member who can be contacted in the event the participant relocates. Our regular phone contact will help participants feel connected to and invested in the study.

**C.9. Sample size and power considerations for primary outcomes.** Schmitz et al found baseline insulin in the control group to be 4.46 (+/- 0.59) uU/ml and in intervention subjects to be 5.18 (+/- 0.54) uU/ml in an exercise study.^67^ Over 39 weeks of exercise intervention, insulin increased an average of 1.16 uU/ml in the control group and 0.05 uU/ml in the intervention group, for a net intervention effect of 1.1 uU/ml. Using this data, the sample size to achieve 85% power for the primary comparison of insulin change over 12 months between the intervention and control groups using a Bonferroni correction with two-sided 0.05/2 = 0.025 and an estimated correlation over 39 weeks for insulin of 0.5 is n = 2σ^2^_change_ (Z_0.9875_ + Z_0.85_)^2^/ Δ^2^

where σ^2^_change_ = 2 σ^2^_baseline_ (1 – ρ) = 123.63, σ^2^_baseline_ = 0.59^2^, ρ = 0.5, Z_0.9875_ = 2.24, Z_0.85_ = 1.035, and Δ = 1.1 This yields a sample size of 6 subjects per group.

The estimates of change for insulin are comparable to the difference between the highest and lowest quintiles of physical activity in the Nurses’ Health Study (difference = 1.37 μU/ml) and the Health Professionals’ Follow-up Study (difference = 2.4 μU/ml).^68^ The difference in C-peptide between highest and lowest quintile of physical activity among women in the Nurses’ Health Study was 0.44 ng/mL. The mean level at baseline was 1.92 +/- 0.91 ng/mL. In the Health Professionals Follow-up Study, the within person correlation for C-peptide over four years was 0.57. This yields a sample size of 79 subjects per group. We can assume the within person correlation over one year is higher. If the correlation rises to 0.73, the sample size is 50 subjects per group.

In a 12-week exercise study of lean men, Dekker et al reported a mean IL-6 level of 2.8 +/- 0.6 pg/mL at baseline and of 1.9 pg/mL at study end for a mean change of 0.9 pg/mL.^87^ Assuming a correlation of 0.85, this yields a sample size of 3 subjects per group. If the correlation drops to 0.5, the sample size increases to 10 subjects per group.

Thus, based on our estimates, we estimate that 20 subjects per group is sufficient to detect differences in several of our outcomes, but not all. 50 subjects per group will provide sufficient power to detect differences in all our markers. However, as this is a pilot project we are not concerned with obtaining sufficient power in our outcomes and we will enroll 20 subjects (10 per group). We realize that participant drop out may prohibit our ability to detect differences in some outcomes, but as noted above, **this is a pilot feasibility study**.

**C.11. Data analysis**.

**C.11.a. Primary Aim:** To conduct a dose response pilot trial of low (30 min/day) or high (60 min/day) dose exercise in men and women at increased risk of colon cancer. The major outcomes are changes in serum levels of four risk-related biomarkers: insulin, C-peptide, IL-6 and PGE-2.

Hypothesis 1.1: First we will evaluate randomization success by comparing the two intervention groups on demographic and health factors. We will then evaluate intervention effects by differences in biomarker levels at study end between intervention groups using linear regression. We will compare differences in mean change in each of the biomarkers from baseline to three months between intervention groups. The analysis is based on change over three months using mixed effects regression models with PROC MIXED of SAS. The model is:

Y_it_ = α + β_1_t + β_2_X_i1_ + β_3_X_i1_t + β_4_tY_i0_ + e_it_

where Y_it_ = biomarker for subject i at time t, where t = 0 for baseline, t = 1 for 3 months.

X_i1_ = 1 if i^th^ subject is in the Plus intervention group, = 0 otherwise; e_it_ ~ N(0, σ^2^)

Of primary interest is β_3_ = mean difference in biomarker level change between intervention groups. The coefficient β_2_ allows for mean differences in level between groups at baseline. The coefficient β_4_ allows the change in biomarker to depend on initial level. We will conduct unadjusted analyses because of the randomization design but will also examine the effects of adjusting for sociodemographic factors, and family history of colon cancer. We will conduct outcome analyses based on assigned treatment at randomization regardless of adherence (intent to treat analysis). Throughout our analyses missing data will be imputed.^139^

**C.11.b. Secondary Aim: To compare changes in the secondary outcome of physical activity over three months.**

Hypothesis 2.1: We will evaluate the intervention effectiveness at changing physical activity levels by comparing change in average step count recorded on blinded pedometers from baseline to follow up between the intervention groups. Mixed effects regression will be used to account for the longitudinal design. The outcome variable will be the average steps per day for the measurement period. The measurement period at baseline and follow-up is the seven day period participants were required to wear the blinded pedometer. Only days where the participant wore the pedometer for at least eight hours will be counted and the average will be based on the number of full days of wear. The analysis is based on change using mixed effects regression models similar to that outlined in Aim 1. We will also examine the percentage of intervention participants meeting the 10,000 and 13,000 steps per day goals at the study conclusion based on data recorded on the pedometer. We will use the last seven days of the phase. We will also examine the percent of participants adhering to their study arm prescription (defined as completing 90% of required physical activity) at three months. We will examine whether sociodemographic factors, overall physical activity level, or family history of colon cancer are associated with meeting the intervention step count requirement. Finally, we will examine whether the intervention changed total physical activity levels by comparing baseline and follow-up total physical activity levels (as measured by the PPAQ) in the intervention groups and by comparing change in those levels between the intervention groups using the general estimating equation modification to linear regression.

**D. Data Safety and Monitoring**

Participants will be identified in data records by a study ID code to protect data. Identifiable information will be kept separate from study data and will be locked in a study file cabinet. Electronic files will not contain identifiable participant information. Identifiable information will be destroyed at the end of the study.

All investigator-level staff members have completed the NIH human subject’s certification as required. All study field staff, investigators, and others involved with participants and/or data collection and handling will have completed the NIH human subjects’ certification as required. Human subjects concerns will be a standard agenda item for project meetings. We will participate in a standard continuing review process. All adverse events reported to study staff or investigators will be discussed by the investigators. Investigators will evaluate the potential risk to other participants.

The principal investigator will review all patient data at least every six months, and provide a semi-annual report to the QASM Committee. This report will include

1. the protocol title, IRB protocol number, and the activation date of the study.
2. the number of patients enrolled to date
3. the date of first and most recent patient enrollment
4. a summary of all adverse events regardless of grade and attribution
5. a response evaluation for evaluable patients
6. a summary of any recent literature that may affect the ethics of the study.

The study principal investigator and study coordinator will monitor for toxicities/adverse events on an ongoing basis. Once the principal investigator or study coordinator becomes aware of a serious adverse event, the SAE will be reported to the HRPO and QASM Committee within 10 working days.

**E. References**

**1.** American Cancer Society. *Cancer Facts and Figures 2007.* Atlanta, GA: American Cancer Society; 2007.

**2.** Howe HL, Wingo PA, Thun MJ, et al. Annual report to the nation on the status of cancer (1973 through 1998), featuring cancers with recent increasing trends. *J Natl Cancer Inst.* 2001;93(11):824-842.

**3.** International Agency for Research on Cancer WHO. *IARC Handbooks of Cancer Prevention: Weight Control and Physical Activity, Volume 6.* Lyon, France: International Agency for Research on Cancer; 2002.

**4.** anonymous. Physical activity trends--United States, 1990-1998. *MMWR Morb Mortal Wkly Rep.* 2001;50(9):166-169.

**5.** Haskell WL, Lee IM, Pate RR, et al. Physical Activity and Public Health. Updated Recommendation for Adults From the American College of Sports Medicine and the American Heart Association. *Circulation.* Aug 1 2007.

**6.** Physical Activity Guidelines Advisory Commitee. *Physical Activity Guidelines Advisory Committee Report, 2008.* Washington, DC: U.S. Department of Health and Human Services; 2008.

**7.** Giovannucci E, Ascherio A, Rimm EB, Colditz GA, Stampfer MJ, Willett WC. Physical activity, obesity, and risk for colon cancer and adenoma in men. *Ann Intern Med.* Mar 1 1995;122(5):327-334.

**8.** Gerhardsson M, Norell SE, Kiviranta H, Pedersen NL, Ahlbom A. Sedentary jobs and colon cancer. *Am J Epidemiol.* May 1986;123(5):775-780.

**9.** Gerhardsson M, Floderus B, Norell SE. Physical activity and colon cancer risk. *Int J Epidemiol.* Dec 1988;17(4):743-746.

**10.** Gerhardsson de Verdier M, Steineck G, Hagman U, Rieger A, Norell SE. Physical activity and colon cancer: a case-referent study in Stockholm. *Int J Cancer.* Dec 15 1990;46(6):985-989.

**11.** Martinez ME, Giovannucci E, Spiegelman D, Hunter DJ, Willett WC, Colditz GA. Leisure-time physical activity, body size, and colon cancer in women. Nurses' Health Study Research Group. *J Natl Cancer Inst.* Jul 2 1997;89(13):948-955.

**12.** Lee IM, Manson JE, Ajani U, Paffenbarger RS, Jr., Hennekens CH, Buring JE. Physical activity and risk of colon cancer: the Physicians' Health Study (United States). *Cancer Causes Control.* Jul 1997;8(4):568-574.

**13.** Lee IM, Paffenbarger RS, Jr., Hsieh C. Physical activity and risk of developing colorectal cancer among college alumni. *J Natl Cancer Inst.* Sep 18 1991;83(18):1324-1329.

**14.** Severson RK, Nomura AM, Grove JS, Stemmermann GN. A prospective analysis of physical activity and cancer. *Am J Epidemiol.* Sep 1989;130(3):522-529.

**15.** Fredriksson M, Bengtsson NO, Hardell L, Axelson O. Colon cancer, physical activity, and occupational exposures. A case-control study. *Cancer.* May 1 1989;63(9):1838-1842.

**16.** Whittemore AS, Wu-Williams AH, Lee M, et al. Diet, physical activity, and colorectal cancer among Chinese in North America and China. *J Natl Cancer Inst.* Jun 6 1990;82(11):915-926.

**17.** Slattery ML, Schumacher MC, Smith KR, West DW, Abd-Elghany N. Physical activity, diet, and risk of colon cancer in Utah. *Am J Epidemiol.* Nov 1988;128(5):989-999.

**18.** Slattery ML, Edwards SL, Ma KN, Friedman GD, Potter JD. Physical activity and colon cancer: a public health perspective. *Ann Epidemiol.* Feb 1997;7(2):137-145.

**19.** Samad AK, Taylor RS, Marshall T, Chapman MA. A meta-analysis of the association of physical activity with reduced risk of colorectal cancer. *Colorectal Dis.* May 2005;7(3):204-213.

**20.** Slattery ML. Physical activity and colorectal cancer. *Sports Med.* 2004;34(4):239-252.

**21.** Cronin KA, Krebs-Smith SM, Feuer EJ, Troiano RP, Ballard-Barbash R. Evaluating the impact of population changes in diet, physical activity, and weight status on population risk for colon cancer (United States). *Cancer Causes Control.* May 2001;12(4):305-316.

**22.** Wolin KY, Lee IM, Colditz GA, Glynn RJ, Fuchs C, Giovannucci E. Leisure-time physical activity patterns and risk of colon cancer in women. *Int J Cancer.* Aug 23 2007.

**23.** Brownson RC, Eyler AA, King AC, Brown DR, Shyu YL, Sallis JF. Patterns and correlates of physical activity among US women 40 years and older. *Am J Public Health.* Feb 2000;90(2):264-270.

**24.** Crespo CJ, Keteyian SJ, Heath GW, Sempos CT. Leisure-time physical activity among US adults. Results from the Third National Health and Nutrition Examination Survey. *Arch Intern Med.* Jan 8 1996;156(1):93-98.

**25.** MacDonald RS, Thornton WH, Jr., Bean TL. Insulin and IGE-1 receptors in a human intestinal adenocarcinoma cell line (CACO-2): regulation of Na+ glucose transport across the brush border. *J Recept Res.* 1993;13(7):1093-1113.

**26.** Watkins LF, Lewis LR, Levine AE. Characterization of the synergistic effect of insulin and transferrin and the regulation of their receptors on a human colon carcinoma cell line. *Int J Cancer.* Feb 15 1990;45(2):372-375.

**27.** Koenuma M, Yamori T, Tsuruo T. Insulin and insulin-like growth factor 1 stimulate proliferation of metastatic variants of colon carcinoma 26. *Jpn J Cancer Res.* Jan 1989;80(1):51-58.

**28.** Giovannucci E. Insulin and colon cancer. *Cancer Causes Control.* Mar 1995;6(2):164-179.

**29.** Le Marchand L, Wilkens LR, Kolonel LN, Hankin JH, Lyu LC. Associations of sedentary lifestyle, obesity, smoking, alcohol use, and diabetes with the risk of colorectal cancer. *Cancer Res.* Nov 1 1997;57(21):4787-4794.

**30.** La Vecchia C, D'Avanzo B, Negri E, Franceschi S. History of selected diseases and the risk of colorectal cancer. *Eur J Cancer.* 1991;27(5):582-586.

**31.** Will JC, Galuska DA, Vinicor F, Calle EE. Colorectal cancer: another complication of diabetes mellitus? *Am J Epidemiol.* May 1 1998;147(9):816-825.

**32.** Hu FB, Manson JE, Liu S, et al. Prospective study of adult onset diabetes mellitus (type 2) and risk of colorectal cancer in women. *J Natl Cancer Inst.* Mar 17 1999;91(6):542-547.

**33.** Tran TT, Medline A, Bruce WR. Insulin promotion of colon tumors in rats. *Cancer Epidemiol Biomarkers Prev.* Dec 1996;5(12):1013-1015.

**34.** Limburg PJ, Stolzenberg-Solomon RZ, Vierkant RA, et al. Insulin, glucose, insulin resistance, and incident colorectal cancer in male smokers. *Clin Gastroenterol Hepatol.* Dec 2006;4(12):1514-1521.

**35.** Schoen RE, Tangen CM, Kuller LH, et al. Increased blood glucose and insulin, body size, and incident colorectal cancer. *J Natl Cancer Inst.* Jul 7 1999;91(13):1147-1154.

**36.** Yoshida I, Suzuki A, Vallee M, et al. Serum insulin levels and the prevalence of adenomatous and hyperplastic polyps in the proximal colon. *Clin Gastroenterol Hepatol.* Oct 2006;4(10):1225-1231.

**37.** Schoen RE, Weissfeld JL, Kuller LH, et al. Insulin-like growth factor-I and insulin are associated with the presence and advancement of adenomatous polyps. *Gastroenterology.* Aug 2005;129(2):464-475.

**38.** Keku TO, Lund PK, Galanko J, Simmons JG, Woosley JT, Sandler RS. Insulin resistance, apoptosis, and colorectal adenoma risk. *Cancer Epidemiol Biomarkers Prev.* Sep 2005;14(9):2076-2081.

**39.** Wei EK, Ma J, Pollak MN, et al. A prospective study of C-peptide, insulin-like growth factor-I, insulin-like growth factor binding protein-1, and the risk of colorectal cancer in women. *Cancer Epidemiol Biomarkers Prev.* Apr 2005;14(4):850-855.

**40.** Ma J, Giovannucci E, Pollak M, et al. A prospective study of plasma C-peptide and colorectal cancer risk in men. *J Natl Cancer Inst.* Apr 7 2004;96(7):546-553.

**41.** Kaaks R, Toniolo P, Akhmedkhanov A, et al. Serum C-peptide, insulin-like growth factor (IGF)-I, IGF-binding proteins, and colorectal cancer risk in women. *J Natl Cancer Inst.* Oct 4 2000;92(19):1592-1600.

**42.** Otani T, Iwasaki M, Sasazuki S, Inoue M, Tsugane S. Plasma C-peptide, insulin-like growth factor-I, insulin-like growth factor binding proteins and risk of colorectal cancer in a nested case-control study: the Japan public health center-based prospective study. *Int J Cancer.* May 1 2007;120(9):2007-2012.

**43.** Wei EK, Ma J, Pollak MN, et al. C-peptide, insulin-like growth factor binding protein-1, glycosylated hemoglobin, and the risk of distal colorectal adenoma in women. *Cancer Epidemiol Biomarkers Prev.* Apr 2006;15(4):750-755.

**44.** O'Dwyer PJ, Eckhardt SG, Haller DG, et al. Priorities in colorectal cancer research: recommendations from the Gastrointestinal Scientific Leadership Council of the Coalition of Cancer Cooperative Groups. *J Clin Oncol.* Jun 1 2007;25(16):2313-2321.

**45.** Baron JA, Cole BF, Sandler RS, et al. A randomized trial of aspirin to prevent colorectal adenomas. *N Engl J Med.* Mar 6 2003;348(10):891-899.

**46.** Sandler RS, Halabi S, Baron JA, et al. A randomized trial of aspirin to prevent colorectal adenomas in patients with previous colorectal cancer. *N Engl J Med.* Mar 6 2003;348(10):883-890.

**47.** Friedenreich CM, Orenstein MR. Physical activity and cancer prevention: etiologic evidence and biological mechanisms. *J Nutr.* Nov 2002;132(11 Suppl):3456S-3464S.

**48.** Rigas B, Goldman IS, Levine L. Altered eicosanoid levels in human colon cancer. *J Lab Clin Med.* Nov 1993;122(5):518-523.

**49.** Rhodes JM, Campbell BJ. Inflammation and colorectal cancer: IBD-associated and sporadic cancer compared. *Trends Mol Med.* Jan 2002;8(1):10-16.

**50.** Rose-John S, Scheller J, Elson G, Jones SA. Interleukin-6 biology is coordinated by membrane-bound and soluble receptors: role in inflammation and cancer. *J Leukoc Biol.* Aug 2006;80(2):227-236.

**51.** Atreya R, Neurath MF. Involvement of IL-6 in the pathogenesis of inflammatory bowel disease and colon cancer. *Clin Rev Allergy Immunol.* Jun 2005;28(3):187-196.

**52.** Landi S, Moreno V, Gioia-Patricola L, et al. Association of common polymorphisms in inflammatory genes interleukin (IL)6, IL8, tumor necrosis factor alpha, NFKB1, and peroxisome proliferator-activated receptor gamma with colorectal cancer. *Cancer Res.* Jul 1 2003;63(13):3560-3566.

**53.** Chung YC, Chang YF. Serum interleukin-6 levels reflect the disease status of colorectal cancer. *J Surg Oncol.* Aug 2003;83(4):222-226.

**54.** Makino T, Noguchi Y, Yoshikawa T, Doi C, Nomura K. Circulating interleukin 6 concentrations and insulin resistance in patients with cancer. *Br J Surg.* Dec 1998;85(12):1658-1662.

**55.** Koivisto VA, Yki-Jarvinen H, DeFronzo RA. Physical training and insulin sensitivity. *Diabetes Metab Rev.* 1986;1(4):445-481.

**56.** Holt HB, Wild SH, Wareham N, et al. Differential effects of fatness, fitness and physical activity energy expenditure on whole-body, liver and fat insulin sensitivity. *Diabetologia.* Aug 2007;50(8):1698-1706.

**57.** Weiss EP, Holloszy JO. Improvements in body composition, glucose tolerance, and insulin action induced by increasing energy expenditure or decreasing energy intake. *J Nutr.* Apr 2007;137(4):1087-1090.

**58.** Hasson RE, Freedson PS, Braun B. Postexercise insulin action in African-American women. *J Natl Med Assoc.* Nov 2006;98(11):1832-1839.

**59.** Weiss EP, Racette SB, Villareal DT, et al. Improvements in glucose tolerance and insulin action induced by increasing energy expenditure or decreasing energy intake: a randomized controlled trial. *Am J Clin Nutr.* Nov 2006;84(5):1033-1042.

**60.** O'Donovan G, Kearney EM, Nevill AM, Woolf-May K, Bird SR. The effects of 24 weeks of moderate- or high-intensity exercise on insulin resistance. *Eur J Appl Physiol.* Dec 2005;95(5-6):522-528.

**61.** Schneider SH, Amorosa LF, Khachadurian AK, Ruderman NB. Studies on the mechanism of improved glucose control during regular exercise in type 2 (non-insulin-dependent) diabetes. *Diabetologia.* May 1984;26(5):355-360.

**62.** Burstein R, Polychronakos C, Toews CJ, MacDougall JD, Guyda HJ, Posner BI. Acute reversal of the enhanced insulin action in trained athletes. Association with insulin receptor changes. *Diabetes.* Aug 1985;34(8):756-760.

**63.** Wang JT, Ho LT, Tang KT, Wang LM, Chen YD, Reaven GM. Effect of habitual physical activity on age-related glucose intolerance. *J Am Geriatr Soc.* Mar 1989;37(3):203-209.

**64.** Lindgarde F, Saltin B. Daily physical activity, work capacity and glucose tolerance in lean and obese normoglycaemic middle-aged men. *Diabetologia.* Feb 1981;20(2):134-138.

**65.** Dowse GK, Zimmet PZ, King H. Relationship between prevalence of impaired glucose tolerance and NIDDM in a population. *Diabetes Care.* Nov 1991;14(11):968-974.

**66.** Regensteiner JG, Mayer EJ, Shetterly SM, et al. Relationship between habitual physical activity and insulin levels among nondiabetic men and women. San Luis Valley Diabetes Study. *Diabetes Care.* Nov 1991;14(11):1066-1074.

**67.** Schmitz KH, Ahmed RL, Yee D. Effects of a 9-month strength training intervention on insulin, insulin-like growth factor (IGF)-I, IGF-binding protein (IGFBP)-1, and IGFBP-3 in 30-50-year-old women. *Cancer Epidemiol Biomarkers Prev.* Dec 2002;11(12):1597-1604.

**68.** Pischon T, Hankinson SE, Hotamisligil GS, Rifai N, Rimm EB. Leisure-time physical activity and reduced plasma levels of obesity-related inflammatory markers. *Obes Res.* Sep 2003;11(9):1055-1064.

**69.** Campbell KL, McTiernan A. Exercise and biomarkers for cancer prevention studies. *J Nutr.* Jan 2007;137(1 Suppl):161S-169S.

**70.** Fontana L, Klein S, Holloszy JO. Long-term low-protein, low-calorie diet and endurance exercise modulate metabolic factors associated with cancer risk. *Am J Clin Nutr.* Dec 2006;84(6):1456-1462.

**71.** Frank LL, Sorensen BE, Yasui Y, et al. Effects of exercise on metabolic risk variables in overweight postmenopausal women: a randomized clinical trial. *Obes Res.* Mar 2005;13(3):615-625.

**72.** Wegge JK, Roberts CK, Ngo TH, Barnard RJ. Effect of diet and exercise intervention on inflammatory and adhesion molecules in postmenopausal women on hormone replacement therapy and at risk for coronary artery disease. *Metabolism.* Mar 2004;53(3):377-381.

**73.** Furukawa F, Kazuma K, Kawa M, et al. Effects of an off-site walking program on energy expenditure, serum lipids, and glucose metabolism in middle-aged women. *Biol Res Nurs.* Jan 2003;4(3):181-192.

**74.** Houmard JA, Tanner CJ, Slentz CA, Duscha BD, McCartney JS, Kraus WE. Effect of the volume and intensity of exercise training on insulin sensitivity. *J Appl Physiol.* Jan 2004;96(1):101-106.

**75.** Aldred HE, Hardman AE, Taylor S. Influence of 12 weeks of training by brisk walking on postprandial lipemia and insulinemia in sedentary middle-aged women. *Metabolism.* Mar 1995;44(3):390-397.

**76.** Major GC, Piche ME, Bergeron J, Weisnagel SJ, Nadeau A, Lemieux S. Energy expenditure from physical activity and the metabolic risk profile at menopause. *Med Sci Sports Exerc.* Feb 2005;37(2):204-212.

**77.** Giovannucci E, Rimm EB, Liu Y, Willett WC. Height, predictors of C-peptide and cancer risk in men. *Int J Epidemiol.* Feb 2004;33(1):217-225.

**78.** Allen NE, Appleby PN, Kaaks R, Rinaldi S, Davey GK, Key TJ. Lifestyle determinants of serum insulin-like growth-factor-I (IGF-I), C-peptide and hormone binding protein levels in British women. *Cancer Causes Control.* Feb 2003;14(1):65-74.

**79.** Voskuil DW, Bueno de Mesquita HB, Kaaks R, et al. Determinants of circulating insulin-like growth factor (IGF)-I and IGF binding proteins 1-3 in premenopausal women: physical activity and anthropometry (Netherlands). *Cancer Causes Control.* Dec 2001;12(10):951-958.

**80.** Fung TT, Hu FB, Yu J, et al. Leisure-time physical activity, television watching, and plasma biomarkers of obesity and cardiovascular disease risk. *Am J Epidemiol.* Dec 15 2000;152(12):1171-1178.

**81.** Yore MM, Ham S, Ainsworth B, et al. Reliability and validity of the instrument used in BRFSS to assess physical activity. *Med Sci Sports Exerc.* 2007;39(8):1267-1274.

**82.** Abramson JL, Vaccarino V. Relationship between physical activity and inflammation among apparently healthy middle-aged and older US adults. *Arch Intern Med.* Jun 10 2002;162(11):1286-1292.

**83.** Geffken DF, Cushman M, Burke GL, Polak JF, Sakkinen PA, Tracy RP. Association between physical activity and markers of inflammation in a healthy elderly population. *Am J Epidemiol.* Feb 1 2001;153(3):242-250.

**84.** Ford ES. Does exercise reduce inflammation? Physical activity and C-reactive protein among U.S. adults. *Epidemiology.* Sep 2002;13(5):561-568.

**85.** King DE, Carek P, Mainous AG, 3rd, Pearson WS. Inflammatory markers and exercise: differences related to exercise type. *Med Sci Sports Exerc.* Apr 2003;35(4):575-581.

**86.** Colbert LH, Visser M, Simonsick EM, et al. Physical activity, exercise, and inflammatory markers in older adults: findings from the Health, Aging and Body Composition Study. *J Am Geriatr Soc.* Jul 2004;52(7):1098-1104.

**87.** Dekker MJ, Lee S, Hudson R, et al. An exercise intervention without weight loss decreases circulating interleukin-6 in lean and obese men with and without type 2 diabetes mellitus. *Metabolism.* Mar 2007;56(3):332-338.

**88.** Starkweather AR. The effects of exercise on perceived stress and IL-6 levels among older adults. *Biol Res Nurs.* Jan 2007;8(3):186-194.

**89.** Kohut ML, McCann DA, Russell DW, et al. Aerobic exercise, but not flexibility/resistance exercise, reduces serum IL-18, CRP, and IL-6 independent of beta-blockers, BMI, and psychosocial factors in older adults. *Brain Behav Immun.* May 2006;20(3):201-209.

**90.** MacLennan R, Macrae F, Bain C, et al. Randomized trial of intake of fat, fiber, and beta carotene to prevent colorectal adenomas. *J Natl Cancer Inst.* Dec 6 1995;87(23):1760-1766.

**91.** Baron JA, Beach M, Mandel JS, et al. Calcium supplements for the prevention of colorectal adenomas. Calcium Polyp Prevention Study Group. *N Engl J Med.* Jan 14 1999;340(2):101-107.

**92.** Schatzkin A, Lanza E, Corle D, et al. Lack of effect of a low-fat, high-fiber diet on the recurrence of colorectal adenomas. Polyp Prevention Trial Study Group. *N Engl J Med.* Apr 20 2000;342(16):1149-1155.

**93.** McTiernan A, Yasui Y, Sorensen B, et al. Effect of a 12-month exercise intervention on patterns of cellular proliferation in colonic crypts: a randomized controlled trial. *Cancer Epidemiol Biomarkers Prev.* Sep 2006;15(9):1588-1597.

**94.** Campbell KL, McTiernan A, Li SS, et al. Effect of a 12-month exercise intervention on the apoptotic regulating proteins Bax and Bcl-2 in colon crypts: a randomized controlled trial. *Cancer Epidemiol Biomarkers Prev.* Sep 2007;16(9):1767-1774.

**95.** Ainsworth BE, Leon AS, Richardson MT, Jacobs DR, Paffenbarger RS, Jr. Accuracy of the College Alumnus Physical Activity Questionnaire. *J Clin Epidemiol.* Dec 1993;46(12):1403-1411.

**96.** Masse LC, Ainsworth BE, Tortolero S, et al. Measuring physical activity in midlife, older, and minority women: issues from an expert panel. *J Womens Health.* Feb 1998;7(1):57-67.

**97.** Tudor-Locke CE, Myers AM. Challenges and opportunities for measuring physical activity in sedentary adults. *Sports Med.* Feb 2001;31(2):91-100.

**98.** Schneider PL, Crouter SE, Bassett DR. Pedometer measures of free-living physical activity: comparison of 13 models. *Med Sci Sports Exerc.* Feb 2004;36(2):331-335.

**99.** Freedson PS, Miller K. Objective monitoring of physical activity using motion sensors and heart rate. *Res Q Exerc Sport.* Jun 2000;71(2 Suppl):S21-29.

**100.** Bassett DR, Jr., Ainsworth BE, Swartz AM, Strath SJ, O'Brien WL, King GA. Validity of four motion sensors in measuring moderate intensity physical activity. *Med Sci Sports Exerc.* Sep 2000;32(9 Suppl):S471-480.

**101.** Tudor-Locke CE, Myers AM. Methodological considerations for researchers and practitioners using pedometers to measure physical (ambulatory) activity. *Res Q Exerc Sport.* Mar 2001;72(1):1-12.

**102.** Wilde BE, Sidman CL, Corbin CB. A 10,000-step count as a physical activity target for sedentary women. *Res Q Exerc Sport.* Dec 2001;72(4):411-414.

**103.** Welk GJ, Differding JA, Thompson RW, Blair SN, Dziura J, Hart P. The utility of the Digi-walker step counter to assess daily physical activity patterns. *Medicine and Science in Sports and Exercise.* Sep 2000;32(9 Suppl):S481-488.

**104.** Rooney B, Smalley K, Larson J, Havens S. Is knowing enough? Increasing physical activity by wearing a pedometer. *WMJ : official publication of the State Medical Society of Wisconsin.* 2003;102(4):31-36.

**105.** Croteau KA. A preliminary study on the impact of a pedometer-based intervention on daily steps. *Am J Health Promot.* Jan-Feb 2004;18(3):217-220.

**106.** Moreau KL, Degarmo R, Langley J, et al. Increasing daily walking lowers blood pressure in postmenopausal women. *Medicine and Science in Sports and Exercise.* Nov 2001;33(11):1825-1831.

**107.** Tudor-Locke C, Bassett DR, Jr. How many steps/day are enough? Preliminary pedometer indices for public health. *Sports Med.* 2004;34(1):1-8.

**108.** Yamanouchi K, Shinozaki T, Chikada K, et al. Daily walking combined with diet therapy is a useful means for obese NIDDM patients not only to reduce body weight but also to improve insulin sensitivity. *Diabetes Care.* Jun 1995;18(6):775-778.

**109.** Iwane M, Arita M, Tomimoto S, et al. Walking 10,000 steps/day or more reduces blood pressure and sympathetic nerve activity in mild essential hypertension. *Hypertension Research.* Nov 2000;23(6):573-580.

**110.** Tudor-Locke C. Taking steps toward increased physical activity: Using pedometers to measure and motivate. *President's Council on Physical Fitness and Sport Research Digest.* 2002;3(17):1-8.

**111.** Tudor-Locke C. *Manpo-Kei: The Art and Science of Step Counting*. Victoria, BC: Trafford; 2003.

**112.** Tudor-Locke CE, Myers AM, Rodger NW. Development of a theory-based daily activity intervention for individuals with type 2 diabetes. *Diabetes Educ.* Jan-Feb 2001;27(1):85-93.

**113.** Demark-Wahnefried W, Morey MC, Clipp EC, et al. Leading the Way in Exercise and Diet (Project LEAD): intervening to improve function among older breast and prostate cancer survivors. *Control Clin Trials.* Apr 2003;24(2):206-223.

**114.** Culos-Reed SN, Robinson JL, Lau H, O'Connor K, Keats MR. Benefits of a physical activity intervention for men with prostate cancer. *J Sport Exerc Psychol.* Feb 2007;29(1):118-127.

**115.** Campbell KL, Westerlind KC, Harber VJ, Bell GJ, Mackey JR, Courneya KS. Effects of aerobic exercise training on estrogen metabolism in premenopausal women: a randomized controlled trial. *Cancer Epidemiol Biomarkers Prev.* Apr 2007;16(4):731-739.

**116.** Matthews CE, Wilcox S, Hanby CL, et al. Evaluation of a 12-week home-based walking intervention for breast cancer survivors. *Support Care Cancer.* Feb 2007;15(2):203-211.

**117.** Carmack Taylor CL, Demoor C, Smith MA, et al. Active for Life After Cancer: a randomized trial examining a lifestyle physical activity program for prostate cancer patients. *Psychooncology.* Oct 2006;15(10):847-862.

**118.** Basen-Engquist K, Taylor CL, Rosenblum C, et al. Randomized pilot test of a lifestyle physical activity intervention for breast cancer survivors. *Patient Educ Couns.* Dec 2006;64(1-3):225-234.

**119.** Crouter SE, Schneider PL, Bassett DR, Jr. Spring-levered versus piezo-electric pedometer accuracy in overweight and obese adults. *Med Sci Sports Exerc.* Oct 2005;37(10):1673-1679.

**120.** Melanson EL, Knoll JR, Bell ML, et al. Commercially available pedometers: considerations for accurate step counting. *Prev Med.* Aug 2004;39(2):361-368.

**121.** Ogden CL, Carroll MD, Curtin LR, McDowell MA, Tabak CJ, Flegal KM. Prevalence of overweight and obesity in the United States, 1999-2004. *JAMA.* Apr 5 2006;295(13):1549-1555.

**122.** Jacobs DR, Jr., Ainsworth BE, Hartman TJ, Leon AS. A simultaneous evaluation of 10 commonly used physical activity questionnaires. *Med Sci Sports Exerc.* Jan 1993;25(1):81-91.

**123.** Sallis JF, Pinski RB, Grossman RM, Patterson TL, Nader PR. The development of self-efficiacy scales for health-related diet and exercise behaviors. *Health Education Research.* 1988;3:282-292.

**124.** Sallis JF, Grossman RM, Pinski RB, Patterson TL, Nader PR. The development of scales to measure social support for diet and exercise behaviors. *Prev Med.* Nov 1987;16(6):825-836.

**125.** Davis T, Park I, Covell J, Rizzo L, Cantor D. Health Information National Trends Survey (HINTS 2005): Final report. http://hints.cancer.gov/hints/docs/HINTS2005FinalReport-0523.pdf. Accessed March 22, 2007.

**126.** Bennett GG, McNeill LH, Wolin KY, Duncan DT, Puleo E, Emmons KM. Safe to walk? Neighborhood safety and physical activity among public housing residents. *PLoS Medicine.* in press.

**127.** Cohen S, Kamarck T, Mermelstein R. A global measure of perceived stress. *J Health Soc Behav.* Dec 1983;24(4):385-396.

**128.** McHorney CA, Kosinski M, Ware JE, Jr. Comparisons of the costs and quality of norms for the SF-36 health survey collected by mail versus telephone interview: results from a national survey. *Med Care.* Jun 1994;32(6):551-567.

**129.** Wolinsky FD, Wyrwich KW, Kroenke K, Babu AN, Tierney WM. 9-11, personal stress, mental health, and sense of control among older adults. *J Gerontol B Psychol Sci Soc Sci.* May 2003;58(3):S146-150.

**130.** Sanders-Phillips K. Correlates of health promotion behaviors in low-income Black women and Latinas. *Am J Prev Med.* 1996;12(6):450-458.

**131.** Ashing-Giwa K, Ganz PA, Petersen L. Quality of life of African-American and white long term breast carcinoma survivors. *Cancer.* Jan 15 1999;85(2):418-426.

**132.** Ross C, Mirowsky J. Disorder and Decay: The Concept and Measurement of Perceived Neighborhood Disorder. *Urban Affairs Review.* 1999;34:412-432.

**133.** Ross CE, Reynolds JR, Geis KJ. The Contingent Meaning of Neighborhood Stability for Residents' Psychological Well-Being. *American Sociological Review.* 2000;65:581-597.

**134.** Kohout FJ, Berkman LF, Evans DA, Cornoni-Huntley J. Two shorter forms of the CES-D (Center for Epidemiological Studies Depression) depression symptoms index. *J Aging Health.* May 1993;5(2):179-193.

**135.** Carver CS. You want to measure coping but your protocol's too long: consider the brief COPE. *Int J Behav Med.* 1997;4(1):92-100.

**136.** Tudor-Locke C, Bell RC, Myers AM, et al. Controlled outcome evaluation of the First Step Program: a daily physical activity intervention for individuals with type II diabetes. *Int J Obes Relat Metab Disord.* Jan 2004;28(1):113-119.

**137.** Tudor-Locke CE, Myers AM, Bell RC, Harris SB, Wilson Rodger N. Preliminary outcome evaluation of the First Step Program: a daily physical activity intervention for individuals with type 2 diabetes. *Patient Educ Couns.* May 2002;47(1):23-28.

**138.** Chan CB, Ryan DA, Tudor-Locke C. Health benefits of a pedometer-based physical activity intervention in sedentary workers. *Prev Med.* Dec 2004;39(6):1215-1222.

**139.** Raghunathan TE. What do we do with missing data? Some options for analysis of incomplete data. *Annu Rev Public Health.* 2004;25:99-117.
